# Egg and Soy-Derived Peptides and Hydrolysates: A Review of Their Physiological Actions against Diabetes and Obesity

**DOI:** 10.3390/nu10050549

**Published:** 2018-04-28

**Authors:** Stepheny C. de Campos Zani, Jianping Wu, Catherine B. Chan

**Affiliations:** 1 Department of Physiology, University of Alberta, Edmonton, AB T6G 2R3, Canada; zani@ualberta.ca; 2 Department of Agricultural, Food & Nutritional Science, University of Alberta, Edmonton, AB T6G 2R3, Canada

**Keywords:** diabetes, obesity, egg, soy, peptides, hydrolysate, bioactive peptides

## Abstract

Type 2 diabetes and obesity are two chronic conditions associated with the metabolic syndrome and their prevalences are increasing worldwide. The investigation of food protein-derived bioactive peptides that can improve the pathophysiology of diabetes or obesity while causing minimal side effects is desired. Egg and soy proteins generate bioactive peptides with multiple biological effects, exerting nutritional and physiological benefits. This review focuses on the anti-diabetic and anti-obesity effects of egg- and soy-derived peptides and hydrolysates in vivo and in vitro relevant to these conditions. Studies using the intact protein were considered only when comparing the results with the hydrolysate or peptides. In vivo evidence suggests that bioactive peptides from egg and soy can potentially be used to manage elements of glucose homeostasis in metabolic syndrome; however, the mechanisms of action on glucose and insulin metabolism, and the interaction between peptides and their molecular targets remain unclear. Optimizing the production of egg- and soy-derived peptides and standardizing the physiological models to study their effects on diabetes and obesity could help to clarify the effects of these bioactive peptides in metabolic syndrome-related conditions.

## 1. Introduction

Diabetes is a chronic disease marked by the presence of hyperglycemia that occurs when the pancreas cannot produce enough insulin, or the body cannot effectively use the insulin that is produced. Uncontrolled diabetes can lead to several serious complications such as cardiovascular disease, nephropathy, retinopathy, amputation and nerve damage [1,2]. Well-managed diabetes can reduce the risk of these complications and increase life expectancy [1]. The treatment of diabetes requires long-term self-management and adherence to therapy, but several commonly used drugs can cause side-effects, which could negatively impact adherence [3].

Identifying natural products that can improve the disease state while exerting fewer side-effects is a current research trend. Many studies have explored the potential of biologically active peptides derived from food sources, which could be referred to as functional food ingredients. In the absence of a common definition, The European Commission Concerted Action on Functional Food Science in Europe together with The International Life Sciences Institute Europe published a consensus document and defined functional foods as those that beyond their nutritional value can exert one or more physiological effects in the body in a manner that can improve health/well-being or reduce the risk of diseases [4]. These peptides are produced enzymatically or using fermentation under controlled conditions of pH and temperature.

Two interesting sources of bioactive peptides are hen egg and soy. Egg is relatively cheap, found in almost every country and is nutrient-dense, which means it could be affordable and beneficial to a broad range of the world’s population. Although the physiological effects of egg hydrolysates (EH) and peptides have been tested, the majority of publications investigated the angiotensin converting enzyme (ACE)-inhibitory, antioxidant or anti-inflammatory effects as reviewed by Liu et al. [5]. Soy, which is also protein-dense and broadly available, has been mostly studied regarding its antioxidant and anti-inflammatory actions as reviewed by Masilamani et al. [6]. Since an upregulated renin angiotensin system, inflammation and oxidative stress are observed in obesity and diabetes, which in turn are components of the metabolic syndrome (MetS) along with hypertension, it is of interest to study egg and soy in terms of their anti-diabetic and anti-obesity properties.

This review focuses on the effects of EH and soy hydrolysate (SH) or peptides in improving or preventing type 2 diabetes (T2D) and obesity in vivo or relevant in vitro endpoints such as insulin signaling pathways. The inclusion criteria were studies using egg, egg white (EW), egg yolk or soy in the form of hydrolysate or peptides. Studies where the results of intact protein were compared to the hydrolysate were also included. Only studies using oral administration of the hydrolysate or peptides in vivo were considered.

## 2. Diversity of Bioactive Peptides

Although several processes can be used to generate bioactive peptides, the most common method is enzymatic hydrolysis. Substrate specificity of enzymes generates peptides of different amino acid sequences and can be used to optimize the production of peptides with desired biological effects, as can be seen in Tables 1–4. Nevertheless, the peptides are complex and the use of a purification step following hydrolysis is common [7,8,9]. Alternatively, synthetic production of peptides [10,11] can be used to obtain specific peptides and study their physiological action.

Several groups have used the whole hydrolysate instead of individual peptides when studying their effects [12,13,14,15,16,17,18]. In those cases, effects cannot be attributed to a specific peptide, because the enzymatic hydrolysis can generate a myriad bioactive peptides, raising the possibility that the effect observed could be due to a combination of numerous peptides presented in the hydrolysate. Another variable of enzymatic hydrolysis process is the processing duration, which can impact both the peptide sequences and concentration in the hydrolysate [19].

It is worth mentioning that some of the enzymes used in hydrolysate production are not naturally produced by the human body, such as thermoase [20], flavourzyme [8,19] and neutrase [19], which means the peptides produced may not replicate those generated by the natural digestion process in the human body. Even though some studies used enzymes that are naturally produced in humans, such as pepsin and pancreatin [7,11,15,17] there is also no guarantee that the desired peptides would be produced or stable after further gastrointestinal digestion.

Due to the diversity of peptides obtained after enzymatic hydrolysis, multiple mechanisms of action of the peptides may influence outcomes. The length of the peptides can influence the absorption process in the gut as reviewed by Miner-Williams et al. [21] and specific amino acids can have a greater influence in the interaction with enzymes, for example the regions of interaction between a soy peptide and the enzyme dipeptidyl peptidase-IV (DPP-IV) correlated with the presence of the amino acids glutamine and arginine [11].

There is little evidence that accounts for the mechanisms of action of the peptides and important questions remain unanswered. For instance, are the peptides absorbed intact? Or can they initiate a cascade reaction by binding to receptors in the gut cells? Is the integrity and stability of the peptides after gastrointestinal digestion a requirement for them to exert their physiological effects?

## 3. In Vitro Study of Egg Hydrolysate (EH)/Peptides

A summary of in vitro studies and identified peptide sequences are provided in Table 1. Multiple metabolic pathways in several organs are involved in glucoregulation. One possibility to help manage diabetes is inhibition of intestinal α-glucosidase, which is an effective method to delay carbohydrate absorption [22] and reduce blood glucose concentrations. Peptides obtained after pepsin hydrolysis of egg white (EW) exhibited α-glucosidase IC_50_ values ranging from 365 to 1694 µg/mL [7], while peptides obtained from alcalase hydrolysis of egg yolk yielded IC_50_ values ranging from 23 to 40 µmol/L [22]. Beside α-glucosidase inhibition, the peptides from EW exerted multiple activities, for instance, ACE-inhibitory capacity with IC_50_ ranging from 9 to 27 µg/mL and DPP-IV- inhibitory activity with IC50 from 223 to 1402 µg/mL. The only exception was the peptide YIEAVNKVSPRAGQPF, which did not present either α-glucosidase or DPP-IV inhibitory activity [7]. The results suggest that egg peptides can potentially exert more than one physiological effect. Multiple activities exerted by the egg white hydrolysate (EWH) were found in other studies using cell lines as well [19,20]. EWH obtained with different enzymes (Table 2) exerted concomitantly anti-inflammatory, antioxidant, hypocholesterolemic, DPP-IV- and ACE-inhibitory activity [19]. The EWH derived from pepsin and peptidase-mediated hydrolysis had the highest potential against disorders associated with MetS such as hypertension, obesity and T2D, presenting IC_50_ against DPP-IV of <10 mg protein/mL and against ACE ranging from 47 to 151 µg/mL [19].

In the 3T3-L1 adipocyte cell line, thermoase + pepsin-prepared EWH not only sensitized the cells to insulin action but also mimicked insulin signaling. The EWH stimulated adipocyte differentiation by enhancing peroxisome proliferator associated receptor gamma (PPAR-γ) and CAAT/enhancer binding protein alpha (C/EBPα) expression, which led to enhanced adiponectin release and intracellular lipid accumulation. Moreover, these EWH enhanced the phosphorylation of proteins involved in the insulin signaling pathway, such as extracellular signal regulated kinase 1/2 (ERK 1/2), insulin receptor substrate 1 (IRS-1) and insulin receptor and protein kinase B (AKT) [20]. In adipocytes, the same EWH also presented anti-inflammatory properties by reducing cyclooxygenase-2 (COX-2) expression and C-Jun phosphorylation induced by tumor necrosis factor-α (TNF-α) [20]. Thus, the effect of thermoase + pepsin-prepared EWH in 3T3-L1 cells is exerted via insulin receptor and downstream proteins in the insulin signaling pathway. The adipogenic effect observed was partially mediated by PPAR-γ, because peptides identified in the hydrolysate upregulated PPAR-γ expression in vitro [20]. In macrophages, no effects were observed regarding TNF-α using peptidase or pepsin or flavourzyme EWH, but peptidase-prepared EWH reduced IL-6 after lipopolysaccharides stimulation [19].

An improvement in insulin sensitivity was also observed in a muscle cell line exposed to EW peptides. IRW, a peptide from egg ovotransferrin improved insulin resistance induced by angiotensin-II in skeletal muscle cells [10]. The peptide reversed the impaired insulin signaling and glucose uptake by normalizing phosphorylation of the serine residue in IRS and increasing AKT phosphorylation, which contributed to increased translocation of glucose transporter 4 (GLUT4) to the plasma membrane. It was shown that these effects were exerted partly by reducing angiotensin II type 1 receptor expression and reactive oxygen species (ROS) production [10]. In contrast, IQW and LPK egg white-derived peptides only exhibited antioxidant activity [10].

Although anti-diabetic activity is exerted by specific peptides, others presented low or no activity as antidiabetic agents [7,10,19,22], a fact that was attributed to their different amino acid sequences once they all were tested under the same conditions [10,19]. This fact indicates that the effects observed were due to the presence of specific peptides; however, there is a lack of experiments studying the relationship between the amino acid sequence in the peptides and their actions.

In summary, in vitro studies show that EWH or peptides derived from EW and egg yolk can exert multiple biological activities, including antidiabetic, by inhibiting enzymes such as DDP-IV and α-glucosidase or improving insulin sensitivity or signaling. However, the peptide amino acid sequence is important in determining the peptides’ ability to act as antidiabetic agents. Therefore, there is a need for more in vitro experiments to specifically identify the interaction between the peptides, their amino acid sequence and the targets involved in the insulin signaling pathway.

## 4. In Vivo Studies of Egg White Hydrolysate (EWH)/Peptides

In vivo EWH presents multiple biological activities as demonstrated in Table 2. All these in vivo studies were done in rodents and the specific peptides in EWH were not reported. Although no changes were found in circulating insulin, one study observed a reduction in blood glucose concentration and reduced homeostasis model assessment of insulin resistance (HOMA-IR) with protease-prepared EWH treatment [12]. Another three studies reported no changes in blood glucose levels with EWH obtained from protease and alcalase hydrolysis [13,23,24]. Serum leptin concentrations were not statistically different [23,24], and reduction or no changes were observed in plasma adiponectin levels [12,14].

Alongside enlarged adipose tissue, ectopic fat accumulation can lead to insulin resistance (IR) and consequently T2D. Analysis of the lipid content in liver and muscle, and total body fat percentage in rats showed reduced values after protease- and pepsin-prepared EWH treatment [12,13,14,23]. The steatotic state was improved (reduced size and number of fat vesicles), but no histological changes were seen in the adipose tissue with the pepsin-prepared EWH groups presenting similar adipocyte size as the obese control [14]. Stearoyl-CoA desaturase (SCD) is an enzyme involved in fat synthesis and responsible for converting a saturated fatty acid to its respective unsaturated fatty acid [12]. The SCD index is the ratio between those fatty acids and is related to obesity and insulin resistance. Dietary supplementation with protease-prepared EWH decreased SCD index in serum, muscle and liver in rodents [12,13,23]. Several hypotheses were tested in an attempt to elucidate the mechanisms responsible for reducing fat accumulation; for instance, SCD-1 is an enzyme essential in fat synthesis and because the abundance of lipogenic enzymes such as lipoprotein lipase (LPL) and fatty acids synthase (FAS) were not altered by EWH, the decrease in non-adipose tissue lipid content was attributed to the reduced SCD index [3]. Garcés-Rimon et al. postulate that the reduction in liver fat accumulation could be due to the ability of pepsin-prepared EWH to stimulate FFA oxidation in the hepatocytes but this hypothesis has not been tested [14]. Another possibility is that the reduction in fat accumulation occurred due to increased fat excretion. Indeed, two studies reported increased excretion of cholesterol (CHOL) and/or triglyceride (TG) and total bile acids in feces after protease-prepared EWH treatment [12,23]. In serum, reduction of CHOL or TG and/or free fatty acids (FFA) was observed [13,14,23]. However, no improvement in serum lipid profile was seen in another two studies [12,24]. An interesting corollary finding was that protease-prepared EWH increased muscular mass while decreasing fat accumulation, although the mechanism by which the hydrolysate acts remains unclear [13].

A study of the gut microbiota revealed that pepsin-prepared EWH treatment improved dysbiosis in obese rats; furthermore, short chain fatty acid (SCFA) and lactate concentrations in feces were lower compared to the obese group [15]. SCFAs are produced by gut microbiota through fermentation of dietary fiber, carbohydrates and peptides and are shown to improve glucose homeostasis and insulin sensitivity in rodents [25]; in addition, increased fecal SCFA content is found in obese human subjects [26]. Mechanisms that could explain the lower fecal SCFA in the pepsin-prepared EWH-fed group include maintenance of intestinal microbiota homeostasis or prevention of absorptive dysfunction by EWH; nevertheless, Requena et al. hypothesized that the change in microbiota occurred secondary to peptide absorption, with their actions on target tissues as antioxidant and anti-inflammatory agents leading to modulation of the gut microbiota [15] but there is as yet no evidence for this hypothesis.

Anti-inflammatory and antioxidant activity can contribute towards obesity and diabetes management [15]. In two studies in vivo, treatment with pepsin- and alcalase-prepared EWH reduced TNF-α in plasma and kidney and reduced malondialdehyde levels in plasma and urine indicating antioxidant properties [14,24]; these results are compatible with those observed in vitro previously mentioned in Table 1.

When not treated, diabetes can lead to several complications including nephropathy. Although NWT-03, an alcalase-prepared EWH, exerted in vitro DPP IV-inhibitory activity, in vivo it was not efficient in improving the diabetic state; however, the treatment reduced renal injury development and albuminuria in T2D rats [24]. When compared with vildagliptin (VIL), a currently used DPP-IV inhibitor, both NWT-03 and VIL exerted renal protection effects but only VIL increased GLP-1 levels. Therefore, it is believed that NWT-03 and VIL can act via similar mechanisms but independently of their DPP-IV inhibitory activity [24].

It is worth noting that when administered in a single dose, protease-prepared EWH did not alter lipid profile, inhibit pancreatic lipase or slow food transit [23]. Interestingly, when compared, protease-prepared EWH and EW, both prevented fat accumulation and increased muscle mass, but EW increased fat excretion compared to EWH [13,23].

To summarize, EWH presented antidiabetic properties in vivo, reducing ectopic fat accumulation in liver and muscle, which can enhance insulin sensitivity, and increasing fat excretion, which reduces absorption of calories and could contribute to weight loss. It also protected against diabetes complications (nephropathy), but little or no change was observed regarding blood glucose, adiponectin or insulin levels and regarding inhibition of DPP-IV. The discrepancies in the results observed in vivo could be attributed to the difference in the physiological background of the animals used but is more likely due to variation in the mixture of peptides present in the hydrolysates. Furthermore, the studies suggest that bioactive peptides present in the EWH were responsible for at least part of the effects observed; nevertheless, no measurement of the peptides in plasma, identification of those peptides or any other specific assay was conducted. There is a gap in the literature to explain the mechanism of absorption and action of these peptides.

## 5. In Vitro Studies of Soy Hydrolysates (SH)/Peptides

Soybean also contains bioactive peptides, and eight studies evaluating the in vitro effects of SH or peptides against diabetes and obesity are summarized in Table 3. Similarly to EH, during adipocyte differentiation SH obtained from pepsin hydrolysis increased lipid accumulation, expression of PPAR-γ and the expression and secretion of adiponectin in a dose dependent manner in 3T3-L1 pre-adipocytes; furthermore, this SH enhanced glucose uptake and expression of GLUT4, which could contribute to improve insulin sensitivity [16]. It is believed that this SH stimulated pre-adipocyte differentiation through PPAR-γ activation, although the SH did not present PPAR- γ ligand activity [16]. Interestingly, a study found that compared to pepsin + pancreatin-prepared SH from ungerminated soybeans, using germinated soybean hydrolysate reduced the number of adipocytes during the differentiation process and increased lipolysis in mature adipocytes, which could lead to less fat accumulation [17].

Higher lipolysis in 3T3-L1 mature cells was observed after treatment with flavourzyme-prepared SH even after gastrointestinal (GI)-simulated digestion as well [8]. Along similar lines, SH prepared with alcalase lowered lipid accumulation and downregulated LPL and FAS gene expression (enzymes involved in the lipid uptake and de novo fatty acid synthesis) in the absence of or following GI-simulated digestion [18]. A hydrolysate obtained only with naturally-occurring GI enzymes (pepsin + pancreatin) exerted similar effects, although to a lesser extent. It suggests that GI digestion in vivo may not markedly affect the bioavailability of that SH [18] although whether that is true for all hydrolysates remains to be determined. β-conglycin is a storage protein naturally found in soybean and it is interesting to note that the higher the β-conglycin concentration in the hydrolysate, the higher the inhibition of LPL and FAS [18].

IR in skeletal muscle and liver is a prominent state found in T2D. Pepsin + pancreatin- prepared SH and its fractions (peptides not identified) enhanced glucose uptake in L6-muscle cells; in addition, the fractions, but not the SH, were able to activate AMPK pathway in those cells [9]. Glucose uptake in C2C12 skeletal muscle cells was also enhanced by another soy peptide, named aglycin [27]. In HepG2 cells soy peptides previously known to modulate cholesterol metabolism by activating AMPK and ERK1/2 pathway [28], affected glucose metabolism by enhancing AKT phosphorylation, which in turn inactivated glycogen synthase kinase 3 (GSK3) by phosphorylating its serine residue, which can lead to higher glucose storage [29]. Moreover, the peptides increased glucose uptake and enhanced the expression GLUT4 and GLUT1 in liver cells [28]. One of these peptides (IAVPTGVA) also presented DPP-IV inhibitory activity with an IC_50_ value of 106 µM, and the regions of interaction between IAVPTGVA and DPP-IV were identified as the amino terminal Glu205 and Glu206 and carboxyl terminal Arg358 residues [11].

Inflammation is not the focus of this review, but it is linked with obesity and diabetes. Two studies reported changes in inflammatory markers by SH [18] or soy peptide [30] in co-cultured adipocytes and macrophages such as, reduced COX-2 and inducible nitric oxide synthase protein and lowered nitric oxide and prostaglandin E2 production [18]. The treatment with synthetized soy peptide FLV reduced the production and effect of inflammatory molecules and improved insulin sensitivity in adipocytes (higher IRS-1 and AKT phosphorylation) [30]. The authors showed evidence that peptide transport into 3T3-L1 cells occurred mainly via the peptide transporter PepT2 [30].

Taken together, the in vitro results show that SH obtained after hydrolysis by specific enzymes and some identified peptides can improve insulin sensitivity, inhibit DPP-IV, increase glucose uptake in muscle and liver, and reduce lipid accumulation and inflammation in adipose tissue (Table 3). Some of the studies suggest that the soy peptides can act through AMPK and AKT pathways to modulate glucose metabolism and via PPAR-γ to stimulate adipocyte differentiation. Although one peptide transporter in adipocytes and regions of interaction between soy peptide and DPP-IV was identified [30], there is still a lack of studies regarding the specific interactions between the peptides and enzymes involved in the glucoregulation process and the mechanism of absorption of those peptides.

## 6. In Vivo Studies of Soy Hydrolysate (SH)/Peptides

The tests in vivo described in Table 4 show that SH can modulate glucose metabolism and reduce body weight. Two studies reported reduced blood glucose levels by SH or peptides [27,31]. Similarly to in vitro studies (Table 3), a 37-amino acid soy peptide named aglycin improved muscle glucose uptake by increasing the phosphorylation of insulin receptor, IRS-1 and AKT, and enhancing membrane GLUT4 levels, which contribute to improved insulin sensitivity in T2D mice [27]. In fact, treatment with aglycin led to similar results as those exerted by metformin in oral glucose tolerance (OGTT) and insulin tolerance tests [27]; furthermore, the release of insulin during OGTT was normal in the treated animals and, as expected, abnormal in T2D mouse controls, suggesting that the effect on glucose tolerance was primarily due to enhanced glucose uptake and insulin sensitivity [27]. It is noteworthy that intact aglycin-37 amino acids were found in blood samples from mice, indicating that it is stable after GI digestion and probably absorbed intact [27].

With regards to serum lipid profile and lipid excretion, protease-prepared SH reduced fat accumulation in genetically obese mice, enhanced lipid excretion and improved plasma CHOL levels in diet obese rats [31]. The reduction in fat accumulation could be due to the higher postprandial energy expenditure observed after intake of protease-prepared SH compared to casein [32]; furthermore, the major contributor to enhanced postprandial energy expenditure was increased exogenous carbohydrate oxidation. Although the effect on energy expenditure was not sustained after 24 h, total carbohydrate oxidation continued to be higher in the SH-treated group, perhaps due to higher plasma insulin levels and lower glucose concentrations during the postprandial period or due to lower lipid absorption and increased carbohydrate absorption [32]. No experiments were conducted to substantiate these hypotheses.

Tests in humans have only been done with respect to glucagon and insulin responses after intake of SH or intact soy protein. SH induced a slower response of insulin and glucagon compared to its intact protein and no effect in plasma glucose was observed. The concentration of soy protein or SH administered did not correlate with the increase in plasma levels of insulin but, interestingly, glucagon was sensitive to protein concentration in a dose-dependent manner for both soy groups [33]. Another comparison, in rodents, showed that SH reduced body weight compared to whey isolate (WI) and whey isolate hydrolysate (WIH). In addition, soy intact protein and SH reduced liver and fat pad weight and maintained body protein percentage compared to WIH and WI, respectively [34].

The results in vivo herein, although scarce show that SH and soy peptides can potentially reduce tissue fat accumulation and increase fat excretion. Moreover, the soy peptide aglycin is resistant to GI digestion and can be absorbed intact by mice [27]. SH may also facilitate metabolic flexibility by shifting to carbohydrate utilization [32]. Nevertheless, only a few studies were done to test SH and peptides as antidiabetic agents in vivo and only one identified the peptide responsible for the effects.

## 7. Conclusions

In conclusion, the research so far shows that both egg and soybean can be rich sources of bioactive peptides; furthermore, they can potentially exert multiple physiological activities, including anti-obesity and anti-diabetic effects, which is relevant in the management of MetS. Bioactive peptides can be produced by different methods such as, enzymatically, chemically or by molecular biology. However, there is a huge variability in the methods, consequently generating many different hydrolysates and peptides, as shown in Table 1, Table 2, Table 3 and Table 4. The duration of the hydrolysis process and the enzymes used generate different amino acid sequences, which influence the type and intensity of activity exerted by the peptides. Although an in silico approach may help to investigate the predictability of peptide generation [11], the predictability, purity and cost-effectiveness of each method vary [35]; therefore, optimization of the production process and the identification of amino acid sequences that can potentially act as anti-diabetic agents are still in need.

In addition, the treatment length and the use of animals with different physiological and genetic backgrounds, such as obesity, diabetes and normal physiology contributed to the discrepancies in the results observed in vivo, which indicates the need of a standardized physiological model to better evaluate the activity of the peptides in diabetic states. Despite these limitations, it is clear that a wide variety of peptides from egg and soybean have overlapping biological activities that may be useful in the treatment of diabetes or obesity.

A weak correlation between the studies mentioned in this review and clinical trials could be drawn. Only one clinical trial using egg peptides was identified and it showed that NWT-03, an egg peptide previously mentioned in this review [24], reduced blood pressure in mild-hypertensive subjects [36]. In terms of soy, only data from clinical trials using black soy peptides were found. Black soy peptides reduced 2h-OGTT, weight, fat mass, leptin levels, blood pressure and oxidative stress in Korean subjects [37,38,39]. At this time, no data from clinical trials associating egg hydrolysate/peptides and glucose metabolism were found.

Even though some investigation of the effects of EH and SH on markers of diabetes and obesity have been done, there is still a lack of studies with focus on the mechanisms of absorption and action of the peptides, especially related to their interaction with cellular and molecular targets involved in insulin and glucose metabolism. Therefore, more studies are necessary to elucidate the effect of EH and SH and their real potential as functional food ingredients to be implemented in the management of obesity and T2D.

## Figures and Tables

**Table 1:** In vitro studies of egg-derived hydrolysates/peptides and their effects related to diabetes and obesity.

	Aims	Hydrolysis	Main Findings	Additional Assays	Peptides
**Egg yolk specific peptides** **Enzymatic activity**					
Zambrowicz et al. 2015 [7]	Investigate multiple biological properties of peptides	Pepsin (120 min)	Three out of four peptides ↓ ACE, α-glucosidase and DPP-IV activity. The peptides presented antioxidant and ion chelating activity.	**DPPH - radical scavenging** All peptides tested presented radical scavenging properties (from 1.5 to 2.3 μMTroloxeq/mg)	YINQMPQKSRE YINQMPQKSREA VTGRFAGHPAAQ YIEAVNKVSPRAGQPF
**Egg white specific peptides** **Enzymatic activity**					
Yu et al. 2011 [22]	Investigate the inhibitory activity of hydrolysates against α-glucosidase and α-amylase and identify peptides	Alcalase (180 min)	Peptides from EW ↓ α-glucosidase but not α-amylase.	N/A	**Ovotransferrin** RVPSLM TPSPR DLQGK AGLAPY **Ovalbumin** RVPSL DHPFLF HAGN WIGLF
**Egg white specific peptides** **Cell culture**					
Garcés-Rimon et al. 2016 [19] 264.7 RAW macrophages	Investigate multiple biological properties of related to the metabolic syndrome	Alcalase Flavourzyme Neutrase Trypsin Pepsin Pancreatin Peptidase Promod 144P (0, 2, 4, 8, 12, 24, 36 and 48 h)	Pepsin hydrolysate: ↓ACE. Peptidase hydrolysate: ↓ ROS, CHOL and IL-6.	**Peptidase hydrolysate (24 h)** **Hypocholesterolemic activity** 0.259 ± 0.01 (mmol bound/mg protein) **ORAC test** 1099.9 ± 0.6 (μmol Trolox/g protein)	**Peptidase hydrolysate** **(24 h)** LPDEVSG DDNKVED GVDTKSD IESGSVEQA GGLVVT
				**Pepsin hydrolysate (8 h)** **Hypocholesterolemic activity** 0.154 ± 0.011 (mmol bound/mg protein) **ORAC test** 574.2 ± 4.0 (μmol Trolox/g protein)	**Pepsin hydrolysate (8 h)** FRADHPPL FSL SALAM YQIGL RADHPFL IVF YAEERYPIL YRGGLEPINF RDILNQ ESIINF
Jahandideh et al., 2017 [20] 3T3-F442A Preadipocyte cell culture	Investigate the effect of hydrolysate on differentiation, insulin signaling and inflammation markers in pre-adipocytes	Thermoase (90 min) + Pepsin (180 min)	↑ intracellular lipid accumulation, adiponectin levels. ↑PPAR-γ and C/EBPα. ↑ p-ERK 1/2, p-IRβ and p-IRS-1. ↓ COX-2 and TNF-α -mediated C-Jun phosphorylation.↑ p-AKT after insulin treatment.	↑ PPAR-γ expression in dose-dependent manner with EWH at 2.5, 5 and 10 mg/mL	ERYPIL VFKGL WEKAFKDED QAMPFRVTEQE
Son et al., 2017 [10] Rat L6 myoblasts	Study the effect of specific ACE inhibitory peptides on insulin resistance induced by Ang-II and their mechanisms of action in muscular cells	N/A	IRW prevented the decrease in glucose uptake induced by Ang-II, normalized serine phosphorylation of IRS and GLUT4 expression and ↑ p-AKT. IRW ↓ AT1R, no effect on AT2R; ↓ ROS and NADPH activity. IQW and LPK peptides had anti-oxidant but no other actions.	N/A	**Ovotransferrin** IRW IQW LPK

Abbreviations: ACE, angiotensin converting enzyme; Ang-II, Angiotensin II; DPP IV, Dipeptidyl peptidase IV; EW, Egg white; IRS-1, Insulin receptor substrate 1; IRS, Insulin receptor; IRβ, Insulin receptor β; COX-2, cyclooxygenase 2; PPARγ, peroxisome proliferator associated receptor gamma; C/EBP-α, CAAT/enhancer binding protein alpha; AKT, protein kinase B; ERK1/2, Extracellular signal regulated kinase 1/2; TNF-α, Tumor necrosis factor alpha; DPPH, 1,1-diphenyl-2-picrylhydrazyl; ROS, Reactive oxygen species; CHOL, Cholesterol; IL-6, Interleukin 6; GLUT4, Glucose transporter 4; AT1R, Angiotensin II type 1 receptor; AT2R, Angiotensin II type 2 receptor; ↑ enhanced/stimulated; ↓ reduced/inhibited.

**Table 2:** In vivo studies of egg-derived hydrolysates/peptides and their effects related to diabetes and obesity in rodents.

	Aims	Hydrolysis	Treatment Details	Food Intake and Body Weight (BW)	Blood/Feces/Urine Analysis	Tissue Analysis	Main Findings
**Egg white hydrolysate** **Studies in rodents**							
Wang et al., 2012 [24] Zucker obese rats	Measure effect of hydrolysate NWT-03 on renovascular damage	Alcalase (6 h)	Aqueous NWT-03 (1 g/kg/day) 15 weeks	Food intake—not given BW—no effect	No effect on blood glucose, insulin, HBA1C, cholesterol and FFA levels. ↑ GLP-1 only by VIL URINE: Reduced MDA levels and decreased albuminuria	KIDNEY - ↓ inflammatory interleukins (IL-1β, IL-13) and TNF-α. Improved FGS, ↓ expression of α-SMA and ↑ TXA2R expression.	No changes in the diabetic profile of the rats; renovascular damage ↓ by NWT-03 treatment.
Ochiai and Matsuo 2014 [13] Wistar rats	Investigate the effect of EW and EWH on fat metabolism and TG content in non-adipose tissues	Protease (duration not specified)	Casein (297 g/kg) EWH (394 g/kg) EW (286 g/kg) 8 weeks	Food intake EWH ↓, EW ↓↓ BW EW ↓	EWH vs. casein- ↓ TG, ALP activity and FFA by EWH. EW vs. EWH- ↓ HDL-CHOL, FFA and ↑ total- CHOL by EWH. FECES EWH vs. casein - ↑CHOL excretion by EWH. EW vs. EWH- ↑ TG, TBA and CHOL excretion by EW.	EWH vs. Casein - Similar results in all parameters, except for ↓ fat mass. MUSCLE- ↑ mass; ↓ SCD index, TG content and G6PDH activity. LIVER- ↓CHOL, TG and SCD index (LIVER). EW vs. EWH - Similar results in all parameters, except for ↑ mass and ↓ SCD (MUSCLE) ↓ SCD (LIVER) by EW.	EW and protease EWH ↓ fat in adipose and non-adipose tissues Inhibited enzymes involved in lipogenesis and ↑ muscular mass and lipid excretion.
Ochiai et al., 2014 [12] Goto-Kakizaki rats	Feeding trial with EWH to study fat and glucose diabetic or normal rats	Protease (duration not specified)	Casein (200 g/kg) And EWH (267 g/kg) 6 weeks	Food intake Not different BW ↓ by EWH	Glucose, HOMA-IR, SCD Index - ↓ No difference between any other parameters tested.	MUSCLE - ↓ TG and SCD. LPL, FAS and G6PDH similar. LIVER - TG similar, ↓ SCD index. Liver, adipose tissue and muscle similar weight.	improved blood glucose levels and HOMA-IR, but not insulin secretion. ↓ TG in muscle and ↓ lipid accumulation in tissues.
Wistar rats			Casein (200 g/kg) And EWH (267 g/kg) 6 weeks	Food intake and BW not different	No difference in any of the parameters tested. (glucose, insulin, HOMA-R, HOMA-P, TG, NEFA, TC, HDL-CHOL, non-HDL-CHOL, adiponectin and SCD index)	MUSCLE - ↓ SCD but LPL, FAS and G6PDH similar LIVER - TG similar, ↓ SCD index. Liver, adipose tissue and muscle similar weight	↓ lipid content in muscle.
Garcés-Rimon et al., 2016 [14] Zucker obese rats	Demonstrate the effects of EWH related to obesity, lipid metabolism, inflammation and oxidative stress	Pepsin (8 or 14 h)	Aqueous EWH (750 mg/kg/day) 12 weeks	No difference in food intake and BW regardless of the hydrolysate	↓TNF-α, FFA and adiponectin, MDA. No changes in blood TG and CHOL.	ADIPOSE TISSUE - ↓weight but no changes in histology. LIVER - ↓ steatosis, ↑ GSH. Similar kidney and liver weight. Longer duration of hydrolysis negated effects.	↓ fat accumulation, improved hepatic steatosis and dyslipidemia. ↓ inflammatory and oxidative stress markers in plasma.
Ochiai et al., 2017 [23] Wistar rats	Study the effect of EW and low allergenic EWH on fat accumulation	Protease (duration not specified)	Equicaloric Diets Casein (297 g/kg) EWH (394 g/kg) EW (286 g/kg) 8 weeks	No difference in food intake and body weight between the three groups.	EWH vs. Casein ↓, total CHOL, ALP. Similar glucose, TG, NEFA, HDL-CHOL, non-HDL-CHOL, HOMA-β and insulin. EW vs. EWH Similar results in all parameters. FECES EWH & EW vs. Casein ↑ TG, CHO and TBA	EWH vs. Casein- Similar results in all parameters, except for ↓ weight, TG and NEFA, SCD index (LIVER). ↓ TG (MUSCLE) EWH vs. EW- Similar results in all parameters, except for ↑ G6PDH activity (muscle), SCD (adipose tissue) ↓ FAS (liver) in EWH.	↓ fat accumulation non- adipose tissues, ↓ intestinal absorption of lipid by increasing lipid excretion. Similar results as EW, however EWH was less allergenic
Requena et al., 2017 [15] Zucker obese rats	Observe the effect of EWH on the gut microbiota of rats	Pepsin (8 h)	Aqueous EWH (750 mg/kg/day) 12 weeks	Food intake N/A BW no difference.	FECES ↓ lactate and SCFA. *Lactobacillus/Enterococcus* and *C. leptum* similar to lean control.	N/A	Partially reverted dysbiosis present in Zucker obese rats.

Abbreviations: EWH, Egg white hydrolysate; FFA, free fatty acids; MDA, Malondialdehyde; EW, Egg white; TG, Triglyceride; CHO, Cholesterol; ALP, Alkaline phosphatase; TBA, Total bile acids; SCD, Stearoyl CoA desaturase; NEFA, Non esterified fatty acids; FGS, Focal glomerulosclerosis; AST, Aspartate aminotransferase; ALT, Alanine aminotransferase; G6PDH, Glucose 6-phosphate dehydrogenase; LPL, Lipoprotein lipase; FAS, Fatty acid synthase; TNF-α, Tumor necrosis factor alpha; α-SMA, Anti-a-smooth muscle actin; VIL, Vildagliptin; HOMA-R, homeostasis model assessment of insulin resistance; HOMA-β, Homeostasis model assessment of insulin secretion; GSH, Reduced Glutathione; HBA1C, Glycated hemoglobin A1C; GLP-1, Glucagon like peptide-1; TXA2R, Thromboxane A2 receptor; SCFA, Short chain fatty acids; WK, week; ↑ enhanced/stimulated; ↓ reduced/inhibited.

**Table 3:** In vitro studies of soy-derived hydrolysates (SH)/peptides and their effects related to diabetes and obesity.

	Aims	Hydrolysis	Outcomes	Main Findings	Peptides
**Soy specific peptides** **Enzymatic activity**					
Lammi et al., 2016 [11]	Verify that soy peptide inhibits DPP-IV in vitro and identify the regions of interactions	Pepsin and/or Pancreatin synthetized peptides	Only IAVPTGVA ↓ DPP-IV activity. Regions of interaction were n-terminus Glu205 and Glu206 and c-terminus Arg358; the peptide has a proline flanked by valine in the fourth n-terminal residue, predicts interaction with DPP-IV.	Soy peptide IAVPTGVA ↓ DPP-IV activity in vitro. YVVNPDNDEN and YVVNPDNNEN were inactive against DPP-IV.	IAVPTGVA YVVNPDNDEN YVVNPDNNEN
**Soy specific peptides** **Cell culture**					
Tsou et al., 2013 [8] 3T3-L1 adipocytes	Isolate and identify peptides from soy hydrolysate with lipolytic activity	Flavourzyme 1% (125 min)	Three peptides ↑ glycerol release. After in vitro GI simulated digestion, VHVV capacity was not affected; ILL and LLL had attenuated lipolytic activity.	Soy peptides ↑ lipolysis in 3T3-L1 adipocytes and were little or not affected by GI enzymes.	ILL LLL VHVV
Lammi et al., 2015 [29] Human HepG2 cells	Verify that soy peptides modulate glucose metabolism	Trypsin or pepsin - synthetized peptides	All three peptides ↑ p-AKT, ↓ GSK3 activation, ↑ GLUT 4 and GLUT 1 mRNA, ↑ glucose uptake. IAVPTVGVA > IAVPGEVA > LPYP). IAVPGEVA and IAVPTVGVA ↑ GLUT1 mRNA more; LPYP ↑ GLUT4 mRNA more.	Soy peptides modulate glucose metabolism and ↑ glucose uptake in liver cells by activation of AKT and AMPK pathways.	IAVPGEVA IAVPTGVA LPYP
Kuak et al., 2016 [30] RAW 264.7 macrophages and 3T3-L1 adipocytes	Demonstrate the mechanism of transport of soy peptide into adipocytes and evaluate TNF-α induced inflammation and insulin response	Synthetized peptide	FLV peptide ↓ TNF-α, MCP-1 and IL-6 in co-cultured cell line (macrophages + adipocytes). FLV ↓ TNF-α-induced p- JNK and p-IKK and ↓ degradation of IκBα. TNF-α induced insulin resistance in adipocytes was ameliorated by FLV (↑ p-IRS-1, p-AKT). PepT2 > PepT1 expressed in adipocytes, ↑ by LPS and TNF-α.	FLV is transported into adipocyte cells mainly through PepT2 action and FLV can ↓ the inflammatory and insulin resistant states linked to obesity mainly by ↓ TNF-α induced inflammatory pathways.	FLV
**Soy Hydrolysate** **Cell culture**					
Martinez-Villaluenga et al., 2009 [18] 3T3-L1 adipocytes and RAW 264.7 macrophages	Study the effect of SH on lipid accumulation and inflammation	Alcalase (3 h) **or** Pepsin + Pancreatin (3 h each)	**Alcalase SH** in 3T3-L1 cells: ↓ lipid accumulation, LPL and FAS mRNA. Further GI simulated digestion did not reduce the bioavailability of Alcalase SH; Compared to Pepsin + pancreatin SH, Alcalase SH ↓ LPL and FAS mRNA in a higher extension, before and after GI digestion. **Alcalase SH** in RAW cells: ↓ LPL-induced nitrite formation, iNOS and COX-2 protein expression, PGE2 production. **Pepsin + pancreatin SH** in 3T3-L1 cells: ↓ lipid accumulation, LPL mRNA, but not FAS mRNA.	SH ↓ lipid accumulation and inflammatory marker expression, even after GI simulated digestion. Downregulation of LPL and FAS partially explain mechanism of action. Higher concentration of β-conglycin in the hydrolysate related to higher activity in vitro.	N/A
González-Espinosa de los Monteros et al., 2011 [17] 3T3-L1 adipocytes	Investigate the effect of germinated vs. ungerminated soybean hydrolysate on fat metabolism in adipocytes. Assess the interaction with soy phytochemicals.	Pepsin + Pancreatin (duration not specified)	Concentration > 1 mg/mL ↓ cell viability during differentiation process (10 days incubation), but not during 24 h of exposure. SH with and without phytochemicals ↓ lipogenesis, with higher germination time correlated to greater lipogenesis reduction. Lipolysis were present in a dose-dependent manner only with SH without phytochemicals treatment.	Germination changed the amino acids composition in the SH and interfered with the responses. Overall, SH ↓ the number of adipocytes during the differentiation process and ↑ lipolysis in mature adipocytes.	N/A
Goto et al., 2013 [16] 3T3-L1 pre-adipocytes	Observe effects of soybean peptic hydrolysate on adipocyte differentiation	Peptic hydrolysate (duration and enzymes not specified)	**During adipocyte differentiation** SH dose-dependently ↑ lipid accumulation, aP2 mRNA, adiponectin mRNA and secretion, PPAR-γ mRNA and protein expression, glucose uptake, GLUT4 mRNA.	SH ↑ adipocyte differentiation via PPAR-γ pathway and ↑ glucose uptake during differentiation process.	N/A
Roblet et al., 2014 [9] L6-skeletal muscle cells	Verify the potential of EDUF to concentrate soy peptides and identify the mechanism of action of those peptides	Pepsin (45 min) + Pancreatin (120 min)	The initial hydrolysate, anionic and cationic peptides ↑ glucose uptake. Only the peptides ↑ p-AMPK.	Anionic and cationic soy ↑ glucose uptake and AMPK phosphorylation in L6- skeletal muscle cells in vitro.	N/A

Abbreviations: SH, Soy hydrolysate; Ap2, adipocyte fatty acid-binding protein; IRS-1, Insulin receptor substrate 1; COX-2, Cyclooxygenase 2; PPARγ, Peroxisome proliferator associated receptor gamma; AKT, protein kinase B; TNF-α, Tumor necrosis factor alpha; LPL, Lipoprotein lipase; FAS, Fatty acid synthase; GLUT4, Glucose transporter 4; GLUT1,glucose transporter 1; SH, Soy hydrolysate; GI- gastrointestinal; iNOS, Inducible nitric oxide synthase; PGE2, Prostaglandin E2; AMPK, Activated protein kinase; JNK, c-Jun N-terminal kinase; IKK, IκB kinase; PepT2, Peptide transporter 2; PepT1, Peptide transporter 1; IL-6, Interleukin 6; DPP-IV, Dipeptidyl peptidase IV; MCP-1, Monocyte chemoattractant protein-1; LPS, Lipopolysaccharide; GSK3, Glycogen synthase kinase 3; ↑ enhanced/stimulated; ↓ reduced/inhibited.

**Table 4:** In vivo studies of soy-derived hydrolysates/peptides and their effects related to diabetes and obesity.

	Aims	Hydrolysis	Treatment Details	Food Intake and Body Weight (BW)	Blood/Feces/Urine Analysis	Tissue Analysis	Main Findings	Peptides
**Soy Specific peptide** **Studies in rodents**								
Lu et al., 2011 [27] BALB/c mice	Investigate effects of soy peptide aglycin as antidiabetic agent	Not specified	HFD + aglycin (50 mg/g) or Metformin (100 mg/kg/d) orally daily for 28 days	No difference in **BW or food intake** (compared with diabetic model control)	Intact peptide detected in plasma after oral administration. **Glucose after 28 days** ↓ by Aglycin. **OGTT and ITT-** Aglycin similar effect as metformin. Insulin release not affected during OGTT.	**Skeletal Muscle ↑** mRNA and total protein of IR and IRS-1. Total AKT and GLUT 4 mRNA not different. ↑ p-IR, p-IRS-1, p-AKT and GLUT4 on membrane.	Aglycin ameliorated glucose intolerance and insulin resistance in T2D mice mainly by ↑ glucose utilization and insulin sensitivity after long-term treatment. **In vitro *-***glucose uptake ↑ in C2C12 skeletal muscle by aglycin in normal and insulin resistant cells.	Aglycin (37 aa)
**Soy hydrolysate** **Studies in rodents**								
Aoyama et al., 2000 [31] Sprague-Dawley rats	Study the effect of soy isolate hydrolysate on weight reduction	Protease (duration not specified)	HFD for 12 weeks + SH (40.4%) or SPI or Casein (39.1%) for 4 weeks	Similar **BW**, **food intake** and body composition in all 3 groups	**SP XSH-** SPIH ↓Glucose, total CHOL and HDL. **SH X Casein-** SH ↓Glucose total CHOL and HDL. **SP X SH**- similar **SH X Casein-** SH ↑ protein and fat % and ↓ apparent fat digestibility	**Liver** SH ↓weight. **fat pad** similar weight	SH ↓ fat accumulation and blood lipid profile levels by ↑ fat excretion. SH ↓ blood glucose in rats.	Mixture of peptides within five to six amino acids in length
Yellow KK mice			HFD for 31 days + SH (40.4%) or Casein (39.1%) for 4 weeks	No difference in **BW.** SH ↓ % fat and ↑ % protein (body composition).	N/A	**Liver** similar weight. **Fat pad** SPIH ↓ weight	SH ↓ fat accumulation and ↑ total protein % in genetically obese KK mice.	
Aoyama et al., 2000 [34] Yellow KK mice	Study the effect of intact soy protein and hydrolysate as anti-obesity agents	Protease (duration not specified)	HFD for 4 weeks + SPI or SPIH or WI or WIH for 2 weeks (energy restricted diet)	SH ↓ **BW** and carcass weight than WI and WIH. **SP and SH** ↓ fat %. **Food intake** similar.	Glucose and TG similar between four groups. SP ↓ total-CHOL than WIH	SP and SH ↓ liver weigh than WIH and WI and ↓ fat pad than WI	No differences were observed between the SP and SH groups; however, compared to WI and WIH. SH ↓weight gain, liver and fat pad weight while maintaining body protein.	N/A
Ishihara et al., 2003 [32] Yellow KK mice	Investigate the effect of soy isolate hydrolysate on energy expenditure	Protease (duration not specified)	HFD for 28 days + high protein diet SH (404 g/kg) or Casein (391 g/kg) for 4 weeks	No difference in **BW or food intake**	SH ↑ lipid content	SH ↓ kidney weight. No difference in liver, muscle, fat pad, heart or spleen weights.	SH- ↑ postprandial energy expenditure, ↑ exogenous carbohydrate oxidation. No difference in postprandial exogenous lipid oxidation. 24-h energy expenditure similar; ↑ 24-h carbohydrate oxidation. SH excreted more TG in feces than casein group.	Mixture of peptides within five to six amino acids in length
**Soy hydrolysate** **Studies in Humans**								
Claessens et al., 2008 [33] Male, non-obese human (average 28 years, BMI 24 kg/m^2^)	Compare glucagon and insulin response after ingestion of soy protein and SH	Not specified	Cross-over trial: consumed drinks containing 0.3, 0.4 or 0.6 g/kg BW of soy protein or SH	N/A	Intact soy protein > SH for insulin and glucagon response. Blood glucose not different. Enhanced effect on glucagon response with increased protein load during intact and SH ingestion than on insulin response.	N/A	Intact soy protein induced a more rapid insulin and glucagon response than the SH. Glucagon was more sensitive to protein load than insulin and responded in a dose dependent manner. No effects in blood glucose were observed.	N/A

Abbreviations: IRS, Insulin receptor; IRS-1, Insulin receptor substrate 1; AKT, protein kinase B; GLUT4, Glucose transporter 4; SPIH, Soy hydrolysate; SP, intact soy protein; HFD, High fat diet; CHOL-cholesterol; TG, Triglyceride; OGTT, Oral glucose tolerance test; ITT, Insulin tolerance test; T2D, Type 2 diabetes; BW, Body weight; BMI, Body mass index; WK, week; ↑ enhanced/stimulated; ↓ reduced/inhibited.
